# Clofibrate Treatment Decreases Inflammation and Reverses Myocardial Infarction-Induced Remodelation in a Rodent Experimental Model

**DOI:** 10.3390/molecules24020270

**Published:** 2019-01-12

**Authors:** Luz Ibarra-Lara, María Sánchez-Aguilar, Elizabeth Soria-Castro, Jesús Vargas-Barrón, Francisco J. Roldán, Natalia Pavón, Juan C. Torres-Narváez, Luz G. Cervantes-Pérez, Gustavo Pastelín-Hernández, Alicia Sánchez-Mendoza

**Affiliations:** 1 Department of Pharmacology, National Institute of Cardiology Ignacio Chávez, Juan Badiano No.1, Col. Sección XVI, Tlalpan, Z.C., Mexico City 14080, Mexico; luzibarralara@gmail.com (L.I.-L.); msanchezaguilar@gmail.com (M.S.-A.); pavitonat@yahoo.com.mx (N.P.); juancarlostn63@hotmail.com (J.C.T.-N.); lgrace22@gmail.com (L.G.C.-P.); pastelingustavo@gmail.com (G.P.-H.); 2 Department of Pathology, National Institute of Cardiology Ignacio Chávez, Juan Badiano No.1, Col. Sección XVI, Tlalpan, Z.C., Mexico City 14080, Mexico; elizabethsoria824@gmail.com; 3 Department of Haemodynamics, National Institute of Cardiology Ignacio Chávez, Juan Badiano No.1, Col. Sección XVI, Tlalpan, Z.C., Mexico City 14080, Mexico; ecovarjes@gmail.com (J.V.-B.); roldan@cardiologia.org.mx (F.J.R.)

**Keywords:** Myocardial infarction, inflammation, ventricular remodeling, reversion of damage, clofibrate, PPARα

## Abstract

Myocardial infarction (MI) initiates an inflammatory response that promotes both beneficial and deleterious effects. The early response helps the myocardium to remove damaged tissue; however, a prolonged later response brings cardiac remodeling characterized by functional, metabolic, and structural pathological changes. Current pharmacological treatments have failed to reverse ischemic-induced cardiac damage. Therefore, our aim was to study if clofibrate treatment was capable of decreasing inflammation and apoptosis, and reverse ventricular remodeling and MI-induced functional damage. Male Wistar rats were assigned to (1) Sham coronary artery ligation (Sham) or (2) Coronary artery ligation (MI). Seven days post-MI, animals were further divided to receive vehicle (V) or clofibrate (100 mg/kg, C) for 7 days. The expression of IL-6, TNF-α, and inflammatory related molecules ICAM-1, VCAM-1, MMP-2 and -9, nuclear NF-kB, and iNOS, were elevated in MI-V. These inflammatory biomarkers decreased in MI-C. Also, apoptotic proteins (Bax and pBad) were elevated in MI-V, while clofibrate augmented anti-apoptotic proteins (Bcl-2 and 14-3-3ε). Clofibrate also protected MI-induced changes in ultra-structure. The ex vivo evaluation of myocardial functioning showed that left ventricular pressure and mechanical work decreased in infarcted rats; clofibrate treatment raised those parameters to control values. Echocardiogram showed that clofibrate partially reduced LV dilation. In conclusion, clofibrate decreases cardiac remodeling, decreases inflammatory molecules, and partly preserves myocardial diameters.

## 1. Introduction

The study of myocardial infarction has gained considerable relevance due to its high incidence and the severe consequences to health and economics. The damage caused during and after myocardial infarction includes immediate and late consequences. In an early stage, there are apparent metabolic changes, cell death, and a beneficial inflammatory response [1]. Later events may include cardiac remodeling, including increased inflammatory state, arrhythmias, heart failure, and even death [2]. Several studies have reported the relevance of cardiac remodeling and inflammation to determine the fate of the heart under ischemic circumstances [3,4,5].

Inflammation acts in the ischemic myocardium as a double-edged sword. In the first minutes after injury, in the ischemic zone there is an important augment in the synthesis and release of proinflammatory cytokines, such as tumor necrosis factor alpha (TNF-α), interleukin-6 (IL-6), interleukin 1-beta (IL-1β), and transforming growth factor 1-beta (TGF-1β). This acute release of cytokines could regulate the survival or apoptosis of myocytes in the infarcted zone. This significant upregulation of proinflammatory cytokines can extend to non-infarcted zones and triggers a second phase of elevated levels of cytokines that promotes interstitial fibrosis and collagen deposition in the contralateral non-infarcted myocardium, leading to a dysfunctional ventricle [6,7,8]. Other factors involved in the long-term damage are toll-like receptors (TLRs), intercellular and vascular cellular adhesion molecules (ICAM and VCAM), matrix metalloproteinases (MMPs), and nuclear factor-kB (NF-kB), among others [9,10].

Cardiac myocytes may die by necrosis, apoptosis, or autophagia depending on the pathways being activated, [11] further contributing to myocardial damage. Therefore, the resolution of the inflammatory process is highly recommendable. Currently, it is known that after myocardial ischemia, necrosis, and augmented ventricular mechanical stress, an inflammatory response tends to generalize [12,13]. Among the adaptative responses elicited by ischemia are cardiomyocyte hypertrophy, cell death, and a change in distribution and composition of the extracellular matrix of cardiac cells [14]. In particular, a rise in collagen that produces fibrosis on ischemic and non-ischemic areas. All those events lead to systolic and diastolic ventricular dysfunction or ventricular remodeling, leading to heart failure. 

In spite of the clear beneficial consequences of reducing inflammation, anti-inflammatory therapies have been applied with no evident improvement in cardiac condition. Pharmacological treatments include non-steroidal anti-inflammatory drugs (NSAIDs) and glucocorticoids [15,16]. Despite the proven anti-inflammatory effect of glucocorticoids, it has been reported that their employment could be detrimental due to secondary effects like volume retention, edema, hyperglycemia, and muscular atrophy, preventing its employment in patients with myocardial infarction [17]. The prescription of commonly used NSAIDs has been limited due to the delayed appearance of ischemic necrosis and imminent risk of developing aneurism and rupture [18].

Peroxisome proliferator-activated receptor (PPAR) agonists have been reported to exert an anti-inflammatory effect. The first evidence was provided by [19], who showed that PPARα^-/-^ mice exhibit higher and longer inflammatory episodes compared to wild type counterparts. In human dyslipidemia, the administration of fibrates, agonists of PPARα isoform, reduced the production of cytokines such as TNF-α, INF-γ, IL-6, and the acute phase inflammatory protein C reactive [20]. Previously, our group reported that clofibrate (100 mg/kg), administered before MI, preserved myocardial viability and prevented overexpression of pro-inflammatory molecules in an experimental model of acute MI [21]. Since most of the experimental studies have treated experimental subjects in a preventive way (before the ischemic maneuver) and limited information regarding treatment of established myocardial infarction exists, the aim of the present study was to evaluate if clofibrate (100 mg/kg) treatment is capable of decreasing inflammation, apoptosis, and reverse ventricular remodeling and myocardial infarction-induced functional damage.

## 2. Results

In order to explore the inflammatory profile of the heart at fourteen days of myocardial infarction, we evaluated the expression of inflammatory markers and inflammatory-induced molecules. 

**Figure 1:**
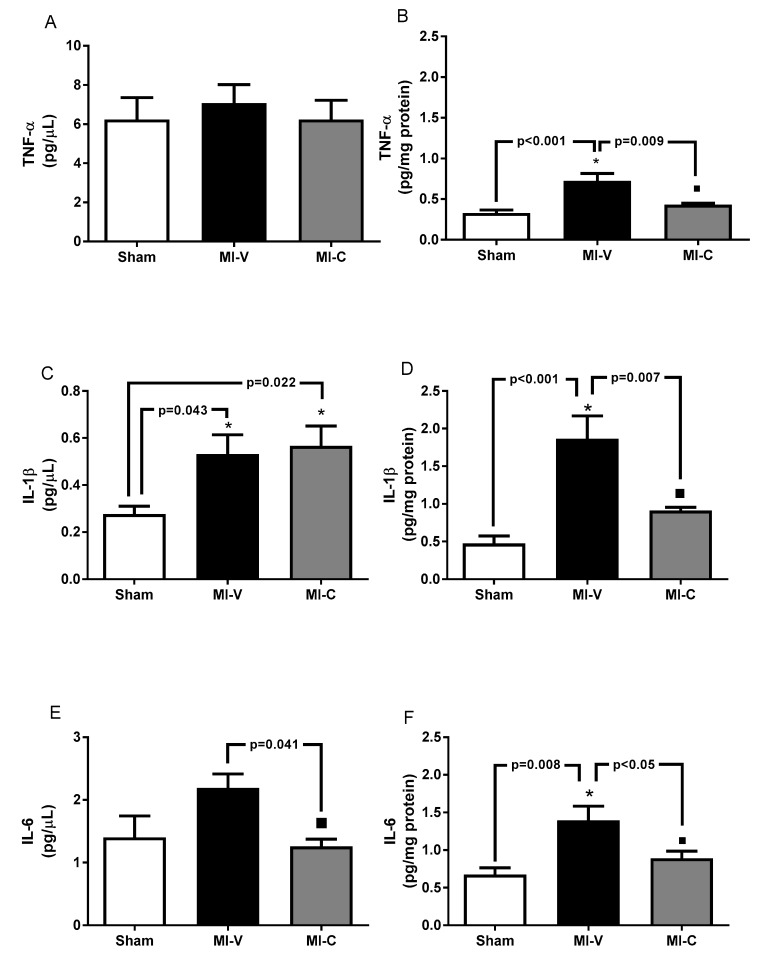
Clofibrate diminished inflammatory cytokines after myocardial infarction (MI). (**A**,**B**) Concentration of TNF-α in serum and left ventricle, respectively. (**C**,**D**) IL-1β concentration in serum and left ventricle, respectively. (**E**,**F**) IL-6 concentration in serum and left ventricle, respectively. Bars represent the mean ± standard error of the mean. White bar represents Sham group, black bar represents MI-V group, and gray bar represents MI-C group; *n* = 6 rats per group, ANOVA-Tukey * *p* < 0.05 vs. Sham and ■ *p* < 0.05 vs. MI-V.

**Figure 2:**
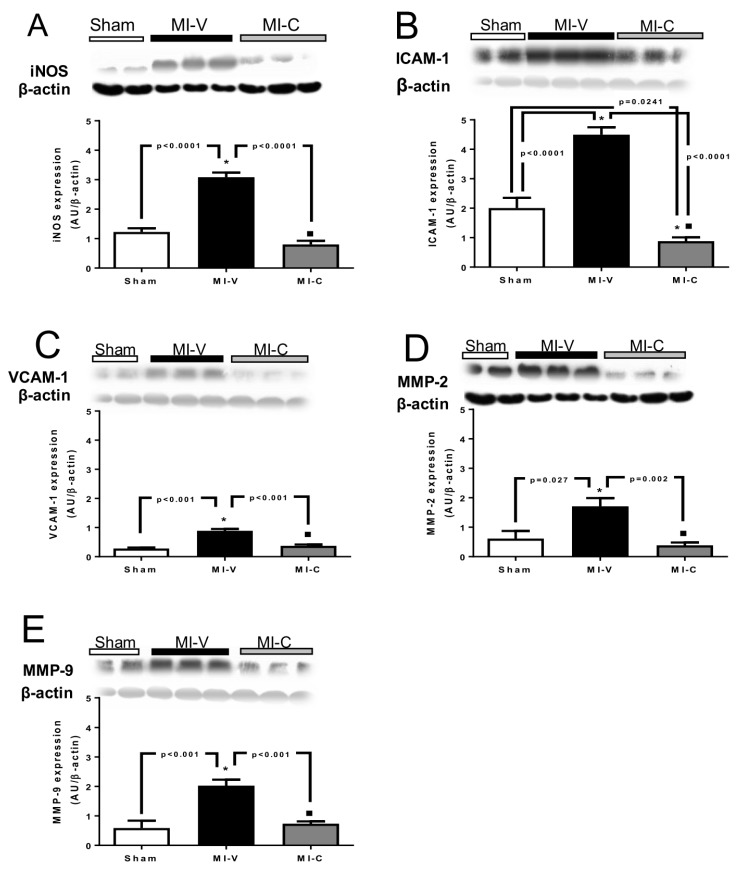
Protein expression of (**A**) iNOS, (**B**) ICAM-1, (**C**) VCAM-1, (**D**) MMP-2, and (**E**) MMP-9 in Sham, myocardial infarction-vehicle (MI-V), and MI clofibrate-treated (MI-C) rats. Each panel shows a representative Western blot and densitometric analysis. β-actin was used as load control and for densitometric normalizing. Image corresponding to β-actin were re-used for illustrative purposes. Bars represent the mean ± standard error; *n* = 6 rats per group, ANOVA-Tukey * *p* < 0.05 vs. Sham and ■ *p* < 0.05 vs. MI-V.

First, we evaluated the expression of IL-6, TNF-α, and IL-1β. Our results indicate that in the serum, there is no change between groups in the concentration of TNF-α, however there is a significant difference in IL-1β and IL-6 between Sham, MI-V, and MI-C (Figure 1A,C,E). We observed that at tissue level, these cytokines increased in the MI-V (*p* < 0.05) group, while they decreased significantly in the group with clofibrate-treatment (Figure 1B,D,F).We evaluated the expression of iNOS in the left ventricle. Our results show that MI induced a higher content of iNOS, an effect that was reverted by clofibrate treatment in MI-C (Figure 2A). Regarding cellular adhesion molecules (ICAM-1 and VCAM-1), our results show that their expression increased in the MI-V left ventricle, and this effect was reversed in rats treated with clofibrate (Figure 2B,C). 

Matrix metalloproteinases participate in the inflammatory pathway, therefore, we evaluated MMP-2 and -9 expressions. Data shows that MI induced a higher expression of both proteins. As expected, clofibrate treatment (7 days) diminished MMPs (2 and 9) protein content in the left ventricles (Figure 2D,E).

**Figure 3:**
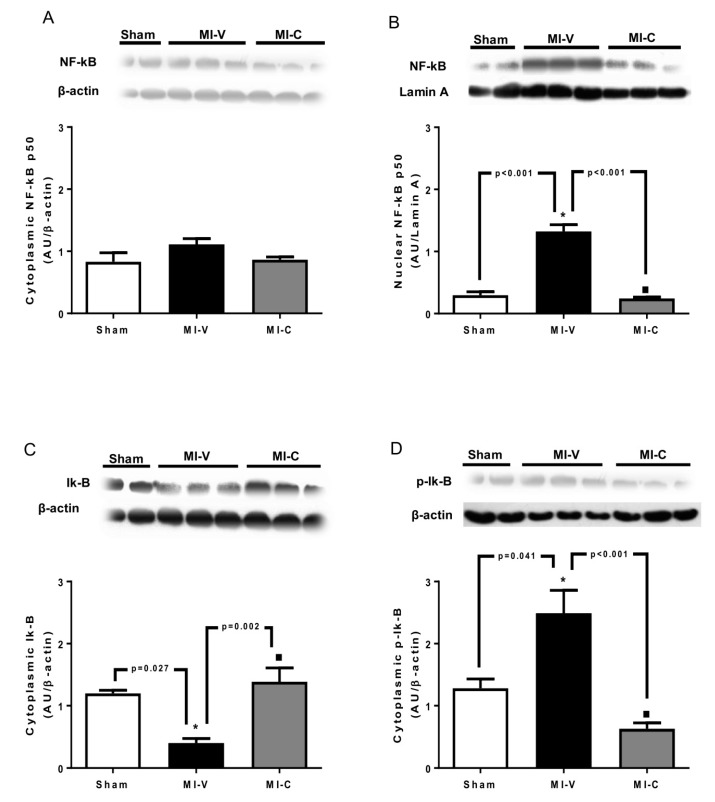
Effect of clofibrate on the activation of NF-kB. The protein expression of (**A**) cytoplasmic NF-kB p50, (**B**) nuclear NF-kB p50, (**C**) cytoplasmic Ik-B, and (**D**) cytoplasmic p-Ik-B was determined in left ventricles from Sham, myocardial infarction (MI)-vehicle treated (MI-V), and MI-clofibrate treated (MI-C) rats. β-actin was used as load control for cytoplasmic and Lamin A for nuclear load control. Images corresponding to β-actin were re-used for illustrative purposes. Bars represent the mean ± standard error; *n* = 6 rats per group, ANOVA-Tukey * *p* < 0.05 vs. Sham and ■ *p* < 0.05 vs. MI-V.

Due to the high relevance of NF-kB modulating the expression of inflammatory-induced molecules, we evaluated its expression. After 14 days of MI, no difference was observed in cytoplasm of the left ventricle among the different groups (Figure 3A). However, the nuclear expression of NF-kB showed a marked increase in MI-V (*p* < 0.05); an event reverted by clofibrate (Figure 3B). In order to explain if the change in nuclear NF-kB was related to Ik-B degradation, we evaluated its expression. Our results show that MI-V exhibits a lower Ik-B cytoplasm content. However, MI rats treated with clofibrate exhibited a higher content of Ik-B in cytoplasm (Figure 3C). These results match with cytoplasmic expression of phospho-Ik-B (p-Ik-B), where its expression in MI-V rats was raised significantly and clofibrate was able to decrease the phosphorylated form in cytoplasm (Figure 3D).

**Figure 4:**
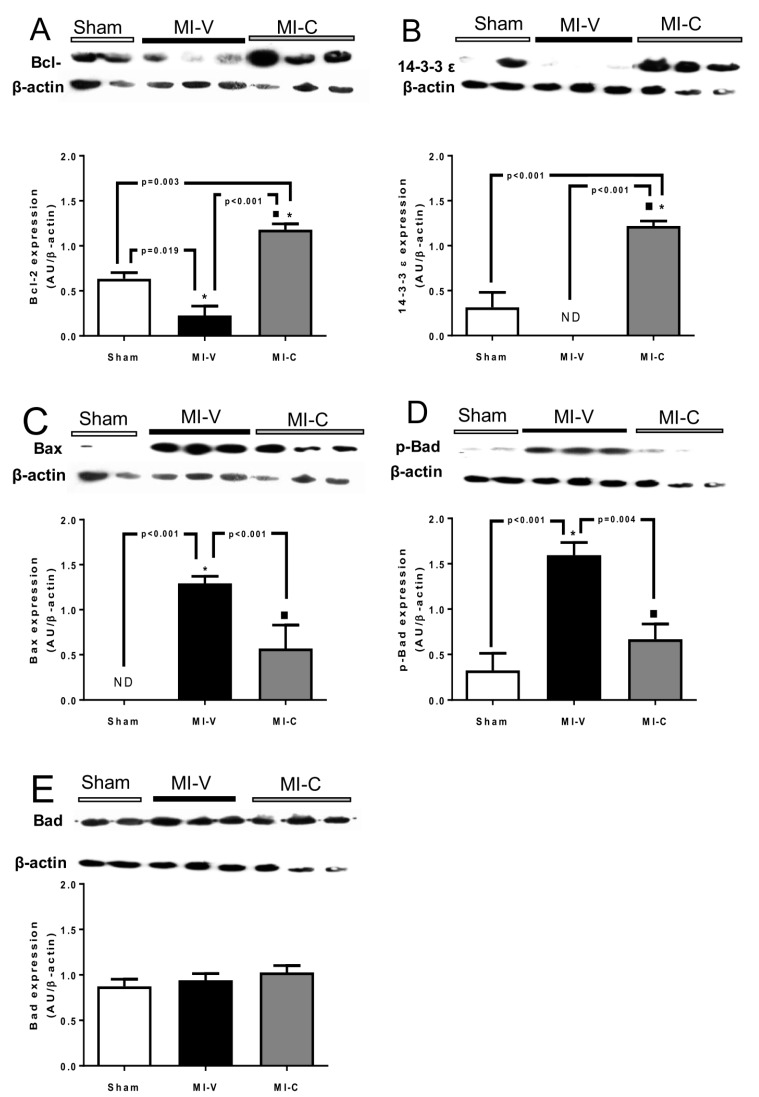
Changes of protein expression on pro- and anti-apoptotic proteins in Sham, myocardial infarction-vehicle treated (MI-V), and MI-clofibrate (MI-C) treated rats. Representative Western blot and densitometric analysis of (**A**) Bcl-2 protein, (**B**) 14-3-3ε protein, (**C**) Bax protein, (**D**) p-Bad protein, and (**E**) Bad protein. β-actin was used as load control and for densitometric normalizing. Images corresponding to β-actin were re-used for illustrative purposes. Bars represent the mean ± standard error; *n* = 6 rats per group, ANOVA-Tukey * *p* < 0.05 vs. Sham and ■ *p* < 0.05 vs. MI-V.

It is well known that inflammatory molecules exert a stimulus on the intrinsic apoptotic pathway. Therefore, we evaluated the expression of apoptotic proteins Bad, p-Bad, and Bax, and antiapoptotic proteins Bcl-2 and 14-3-3ε. As observed after 14 days of MI, the expression of antiapoptotic protein Bcl-2 was decreased and 14-3-3ε expression was not detected (Figure 4A,B). Bax and p-Bad were significantly augmented compared with Sham. Total Bad did not show a significant change in its expression among the different groups. Contrasting with this, clofibrate treatment was able to increase the MI-induced fall in the expression of Bcl-2 and 14-3-3ε and to decrease Bax and p-Bad in the studied left ventricles (Figure 4C,D,E). 

**Figure 5:**
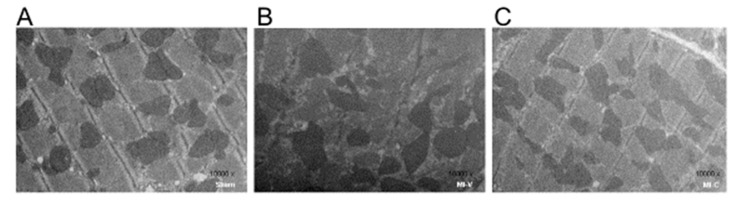
Ultra-microstructure of left ventricle from (**A**) sham, (**B**) myocardial infarction- vehicle (MI-V) treated, and (**C**) myocardial infarction- clofibrate (MI-C) treated rats.

In order to evaluate myocardial infarction-induced tissue damage and the effect of clofibrate, the ultrastructure of the left ventricle was analyzed by transmission electron microscopy (Figure 5). While in Sham rats, the structure of sarcomere exhibits aligned myofilaments, intercalated mitochondria of uniform size and regular morphology, and the Z line clearly defined, in the heart of myocardial infarcted rats, the fibers lost the arrangement and the Z line disappeared. Mitochondria exhibit lower density, become visibly edematous, without defined shape, and crests are lost; the number of mitochondria was increased and became small, round, and were irregularly arranged after MI. Clofibrate treatment partially recovers the sarcomere structure where a Z line appears well defined and mitochondria are visibly intercalated. Other structures such as T-tubules or nucleus are not visible. 

Myocardial functionality in vivo was measured by echocardiographic evaluation. It shows that MI decreased LVEF (*p* < 0.05) in both vehicle- or clofibrate-treated rats. Regarding LV remodeling, data shows that MI induced higher systolic and diastolic diameters. However, clofibrate treatment was able to reduce left ventricular dilatation (*p* < 0.05) (Table 1). Regarding the septum, MI promoted a decreased thickness, independent of the treatment received. Data suggest that no remodelation event or hypertrophy had occurred at 14 days of evolution. However, the increased in systolic and diastolic diameter and the diminished in-septum thickness suggest that the ventricle is not properly pumping the blood, and this is according to the diminution of the ejection fraction. Therefore, 14 days after the infarction, the rats could have developed a dilated myocardiopathy. Clofibrate treatment could reduce the systolic and diastolic diameter, however it could not revert completely, and those changes were not enough to recover the basal ejection fraction. 

**Figure 6:**
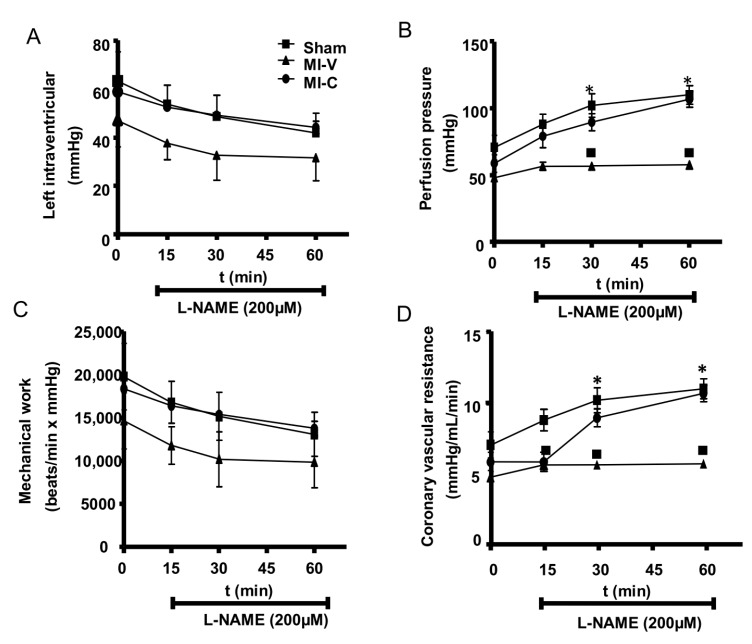
Ex vivo cardiac functioning parameters obtained from Sham, myocardial infarction vehicle (MI-V)- treated, and MI-clofibrate (MI-C)-treated rats. The following parameters were evaluated under basal conditions or in presence of N(ω)-Nitro-L-arginine methyl ester (L-NAME, 200µM): (**A**) Left intraventricular pressure, (**B**) perfusion pressure, (**C**) mechanical work, and (**D**) coronary vascular resistance. Squares represent Sham group, triangles represent MI-V group, and circles represent MI-C group. Bars represent the mean ± standard error. ANOVA * *p* < 0.05 vs. Sham; ■ *p* < 0.05 vs. MI-V; *n* = 3 rats per group.

Ex vivo evaluation of cardiac functioning shows that MI changed the basal left ventricular pressure (LVP) compared to Sham rats (Figure 6). Clofibrate treatment could restore the ventricular pressure. Mechanical work exhibits a comparable behavior among the three groups. Regarding basal perfusion pressure and coronary vascular resistance, we observed no difference between groups, however there is a tendency to reduced basal perfusion pressure in MI rats, however it does not reach significance. When the hearts are buffered in L-NAME perfused conditions, it is remarkable that Sham and clofibrate treated groups had a higher pressure due to the inhibition of NO versus MI-group, suggesting that clofibrate recovers the physiological production of NO, whereas in MI group, the NO is absent. Coronary vascular resistance is calculated in the function of the perfusion pressure and its behavior is similar.

## 3. Discussion

In the present study, we report that treatment with clofibrate for rats with established myocardial infarction is able to decrease late inflammation and partially reverse left ventricular remodeling and functional damage. 

Due to the wide array of pathophysiological factors involved in myocardial infarction-induced remodeling, the treatment to revert this pathology remains elusive [22].

According to Takano et al., the remodeling process starts as a compensatory response to damage and mechanical stress leads to heart failure [23]. This remodeling is produced by the increase in several cytokines: TNF-α, IL-6, IL-1β, and TFG-β1 [7]. These cytokines have been considered the most important in this post infarction process. Our results show that TNF-α, IL-6, and IL-1 are increased in the left ventricle undergoing ischemia for 14 days, thereby increasing the damage. This increase in the production of cytokines after infarction has been demonstrated in various investigations, such as those of Fuchs et al., Tian et al., and Frangogiannis et al. [10,24,25]. 

The wide array of pathophysiological factors involved in the inflammation process as well as their timing of expression adds complexity to the ischemic process. Once neutrophils and monocytes have initiated the reparative process of damaged tissue, the release of ILs, C reactive protein, TNF-α, and MMPs may contribute to further restorative process. However, the excessive or long production of these factors leads to hypertrophy and eventually diastolic dysfunction. In our experimental model of MI, we observed a rise in cellular adhesion molecules (ICAM-1 and VCAM-1), iNOS, and MMPs (2 and 9), considered as inflammation markers, but most important, they can be considered as promoters of further damage. The increase of proinflammatory cytokines brings, as a consequence, a remodelation of cardiac fibers. 

Cellular addition molecules have been reported to promote endothelial leukocyte addition, extravasation to the subendothelial space, and further inflammation. Our results show that on day 14 of myocardial infarction the expression of ICAM-1 is still elevated. The relevance of ICAM-1 promoting further damage has been demonstrated. In patients with stable angina pectoris who developed MI, the levels of soluble serum ICAM-1 were elevated, a clear sign of inflammation [26]. In a rat model of acute myocardial infarction, ICAM-1-blocking antibodies increased the recovery of left ventricular developed pressure and reduced coronary vascular resistance [27]. The risk for the ischemic heart, overexpressing ICAM-1, is reinforced by the parallel raise in the expression of VCAM-1. Proof of this has been reported targeting multiple cell adhesion molecules with RNAi and reducing immune cell recruitment and vascular inflammation after myocardial infarction [28].

Under physiological conditions, iNOS is absent in human hearts, however, proinflammatory cytokines (IL-1β, IL-6, TNF-α, and INF-γ) induce its expression. An increase in iNOS expression and activity, due to cardiac ischemia, decreases cardiac contractility inducing apoptosis and necrosis. There is experimental evidence that indicates that iNOS mRNAs are decreased in PPARα^-/-^ mice, as demonstrated by Paterniti et al. [29]. Our results indicate that clofibrate treatment decreases the expression of iNOS, implying that this would decrease cardiac remodeling as indicated by Kingery et al. [30].

Matrix metalloproteinases are a family of protease enzymes. In the heart, several members of this family have been reported and related to cardiac pathologies. Chen et al. showed that MMP-2 raised 1 week after MI and it reached its highest level 2- and 3-weeks post-MI [31]. Regarding MMP-9, it was demonstrated that its level could be used as a predictor for cardiovascular mortality in patients with coronary artery disease. Our data show that in 14 days post-MI rats the expression of both MMP-2 and -9 raised significantly. These results support those of Chen et al. and provide new evidence regarding clofibrate capability to decrease MMP-2 and -9 expressions in ischemic hearts. The reduction in MMP expression is relevant because extracellular matrix degradation leads to cardiomyocyte slippage and loss of cardiac homeostasis. Further supporting this, it has been reported that the administration of exogenous MMP inhibitors, to MI subjects, improves cardiac remodeling; evaluated as a lower ventricular dilation and a smaller infarct expansion in the late healing phase [32]. In our study, we observed that MI rats treated for 7 days with clofibrate exhibit a lower MI-induced ventricular dilation; an effect resembling that produced by MMP exogenous inhibitors.

In the literature, the phenomenon of apoptosis is characterized by increase in reactive oxygen species and deprivation of oxygen, and its progression was associated with heart failure. Radhiga et al. described in an isoproterenol-induced myocardial ischemia model that Bax, TNF-α, and caspases (3 and 9) were increased as a consequence of ischemia, while Bcl-2 expression was downregulated [33]. In this context, ours results agree with that group. On the other hand, we found that clofibrate augmented the expression of antiapoptotic proteins Bcl-2 and 14-3-3ε, and decreased the expression of proapoptotic proteins Bax and the phosphorylation of Bad. It is known that the inhibition of Bax protects the cells from death, due to a lack of pore formation in the external mitochondrial membrane [34]. Also, clofibrate is capable of raising these anti-apoptotic proteins, protecting the heart from ischemia. In addition, our group previously reported that clofibrate increases the phosphorylation of Akt (Ser 473) [21], a key participant that inactivates Bad apoptotic function and favors cellular survival. 

It is well known that the activation of TLR and IL-1 production leads to the activation of NF-kB, promoting an inflammatory phenotype [35]. Our results show that the treatment with clofibrate decreased the translocation of NF-kB, suggesting that the decrease of inflammatory signals favored the recovery of the infarcted heart.

In the experimental model of MI in rats, the increasing of inflammatory molecules promotes the remodeling process, which is driven by architectural rearrangements of the surviving myocardium, including myocyte hypertrophy, myocardial fibrosis, and ultimately, progressive left ventricular dilation. LV cavity dilatation following myocardial infarction is one of the compensatory reactions of the failing heart. However, excessive dilatation evokes LV systolic and diastolic dysfunction with inevitable impairment of the ejection fraction, which leads to heart failure [36]. In our study, echocardiographic analysis showed that after 14 days of MI progress, the LV diameter at the diastole and systole were increased and ejection fraction was diminished, suggesting the initial process of dilated myocardiopathy. PPARα stimulation with clofibrate diminished the systolic and diastolic diameter but it does not recover ejection fraction. In the study performed by Linz et al. [37], the chronic administration for 8 weeks of AVE8134, a PPARα agonist, to rats after coronary ligation with seven days of progress, improved myocardial contractility, ex vivo relaxation, and cardiac output, as well as a reduction of cardiac hydroxyproline/proline ratio. In our ex vivo study, we observed that after fourteen days of MI, rats elicited a lower LVP and mechanical work, a parameter that was restored in those experimental subjects treated for seven days with clofibrate. The tendency to recover the normal perfusion pressure by clofibrate treatment is likely by keeping the NO production as the L-NAME curves indicate. The NO has an important role in cardio-protection. According to Janssens et al., the inhalation of NO after infarcted or reperfusion injury improves cardiac function, although it fails to reduce the infarct size [38]. 

The ultrastructure analysis allows us to determinate early changes in the myocardial fiber and also associate our echocardiographic or biochemical findings with the structural changes. The ultrastructure analysis includes the observation of muscle fibers and mitochondrial size. As previously reported by Pfeffer and Braunwald, an important marker of myocardial structure injury is mitochondrial arrangement [4]. Clofibrate treatment could restore the form, size, and arrangement of cardiac mitochondria. Clofibrate treatment could reduce the systolic and diastolic diameters, and restore perfusion pressure, most probably through the recovery of ultrastructural myocardial fiber organization. 

We can conclude that the treatment with clofibrate decreases inflammation and reverses myocardial infarction-induced remodeling and functional damage.

## 4. Materials and Methods 

### 4.1. Animals

All animal procedures were conducted in accordance with our Federal Regulations for Animal Experimentation and Care (Ministry of Agriculture, SAGARPA, NOM-062-ZOO-1999, Mexico) and they were approved by the Institutional Animal Care, Use,- and Ethics Committee (INCAR 13-825).

Male Wistar rats (300–350 g) were assigned randomly to one of the following experimental groups: (1) Sham coronary artery ligation (Sham) or (2) Left anterior descending (LAD) coronary artery ligation (MI). Seven days after this procedure, MI rats were further divided to receive vehicle (vegetable oil (V), by intraperitoneal injection (I.P.)) or clofibrate (100 mg/kg (C), (I.P.)) every day for 7 days. Sham rats received V for the same length of time. At the end of the treatment, rats were anaesthetized, and an echocardiogram was conducted. After this, blood was withdrawn, the rats were sacrificed, and the heart was obtained to further analyze protein expression. A second and third set of experimental subjects were assigned for *ex vivo* functional studies (isolated perfused heart) and microscopy, respectively.

### 4.2. Myocardial Ischemia 

Rats anaesthetized with ketamine and xylazine [(80/10) mg/kg intramuscularly (I.M.)] were intubated and artificially ventilated (70 stroke/min, at 8–10 mL/kg). Through the fourth intercostal space, the LAD artery was reached and fully tied (6–0 polypropylene). The chest was sutured by layers and negative pressure was restored [21].

### 4.3. Cytokine Quantification

After 14 days of the ischemic procedure, the left ventricle was carefully separated from the right ventricle, snap-frozen in liquid nitrogen, and powdered. The left ventricular myocardium was homogenized in a lysis buffer (50 mM HEPES, pH 7.5), 150 mM NaCl, 1% glycerol, 1% Triton X-100, 1.5 mM MgCl_2_, and 5 mM EGTA, containing 1 mM phenyl methyl sulfonyl fluoride and a protease inhibitor cocktail. Lysates were clarified by centrifugation at 10,000× *g* and protein concentration was measured by the bicinchoninic acid (BCA) assay (Pierce BCA protein assay kit, Thermo Scientific, Rockford, IL, USA). The values of cytokines were analyzed through a sandwich ELISA method. For serum cytokine study, 100 μL was taken and the same procedure was performed [39,40].

### 4.4. Western Blot

Frozen left ventricles were homogenized in a cocktail of protease inhibitors (Complete ®, Roche Diagnostics, Indianapolis, IN, USA) by means of a polytron (PT-MR2100, Kinematica, Luzern, Switzerland), and protein content was determined by BCA assay (Pierce BCA protein assay kit, Thermo Scientific, Rockford, IL, USA). Eighty micrograms of protein were loaded on acrylamide gel and electrophoretically separated. Proteins were transferred to a polyvinylidene difluoride (PVDF) membrane (0.45 µm Millipore, Billerica, MA, USA) and incubated overnight with polyclonal primary antibodies provided by Santa Cruz Biotechnology (Santa Cruz, CA, USA): ICAM-1, VCAM-1, MMP-2 and -9, IkB, pIkB, NF-kB, iNOS,14-3-3ε, Bad, pBad, and β-actin; Lamin A from Abcam (Cambridge, MA, USA), Bax from BioVision (Mountain View, CA, USA), and Bcl-2 from Invitrogen (Rockford, IL, USA). After washing with PBS, membranes were incubated with appropriate horseradish peroxidase-coupled secondary antibodies. Chemiluminisence was developed using an Immobilion Chemiluminescent Millipore kit (Billerica, MA, USA). Images from films were digitally acquired by means of a digital camera and analyzed using the calibrated densitometer GS-800 USB (Bio-Rad Laboratories, Hercules, CA, USA). β-actin or Lamin A were used as load control [21,41]. 

### 4.5. Nuclear and Cytoplasmic Protein Extraction

Nuclear and cytoplasmic protein fraction contained in 40 mg of ischemic left ventricle were extracted using NE-PER extraction reagents kit (Thermo Scientific, Rockford, IL, USA) following previous reports [21] and manufacturer’s instructions.

### 4.6. Histological Analysis by Electronic Microscopy

Left ventricles from the different experimental groups were fixed in glutaraldehyde (2.5%) and post-fixed in 1% osmium tetroxide in 0.1 M cacodilate buffer. Once fixed, samples were dehydrated and EPON 812 embedded. Ultrathin slices (60 nm thick) were obtained using a Leica Ultracut microtome and mounted on cupper mesh. Slices were contrasted with uranyl acetate and lead citrate before being observed on a JEM- 1011 microscope (JEOL Ltd, Tokyo Japan) at 60 kV [41].

### 4.7. Echocardiogram

At the end of the treatment, rats were anaesthetized (ketamine/xylazine 80/10 mg/kg, I.M.) and subjected to echocardiographic evaluation using a Phillips CX50 Portable General Imaging Ultrasound Machine, equipped with a high frequency 12 MHz transducer (Philips Co., Koninklijke Philips, NV, USA) [42,43]. Two-dimensional guided M-mode echocardiography was performed, and determinations were made from at least 3 beats on each rat. Left ventricular diameters and volumes, ejection fraction (EF), posterior wall, and septal thickness were measured [44,45].

### 4.8. Isolated Perfused Peart

At the end of 7 days clofibrate or V treatment (14 d post MI), rats from the different experimental groups were anaesthetized (ketamine/xylazine 80/10 mg/kg), the heart was excised, and placed in iced cold Krebs buffer. In order to perfuse the heart, it was cannulated retrogradely through the aorta; a constant perfusion volume was applied (15 mL/min). A latex balloon (8–10 mmHg) connected to a transducer was placed into the left ventricle in order to measure left ventricular pressure. The heart was allowed to stabilize (10 min) and a basal set of measurements (left ventricular pressure, coronary vascular resistance, perfusion pressure, and mechanical work) were collected. Finally, Krebs buffer was supplemented with L^ω^-Nitro-L-arginine methyl ester hydrochloride (L-NAME (200 µM)) and cardiac functioning parameters were recorded every 15 min [46].

### 4.9. Data Analysis

Results are expressed as the mean ± standard error of the mean (SEM). For multiple comparisons, we applied one-way analysis of variance (ANOVA). Differences were considered significant when the *p* value was <0.05.

## Figures and Tables

**Table 1:** Echocardiographic changes during MI-V and after clofibrate treatment. Table 1 shows heart rate, left ventricle ejection fraction (LVEF), left ventricle posterior wall thickness, and left ventricle systolic and diastolic diameters. Data are represented as mean ± standard error. ANOVA * *p* < 0.05 vs. Sham; ■ *p* < 0.05 vs. MI-V; *n* = 6 rats per group.

	Sham	MI-V	MI-C
Heart rate (bpm)	276.6 ± 17.33	267.9 ± 21.50	243.3 ± 6.83
LVEF (%)	74.86 ± 2.74	64.71 ± 2.89	66.14 ± 3.03
Posterior wall thickness (mm)	18.57 ± 0.42	19.43 ± 0.57	19.29 ± 0.28
Septum thickness (mm)	18.86 ± 0.76	15.00 ± 1.26 *	14.17 ± 1.07 *
Systolic diameter (mm)	30.14 ± 2.58	46.71 ± 1.47 *	41.00 ± 2.28 *
Diastolic diameter (mm)	59.14 ± 2.81	78.57 ± 1.99 *	70.14 ± 1.94 *^■^
